# Introducing the ICF in the care of individuals with id under consideration of the situation of health care services in poland for individuals with id and mental health problems

**DOI:** 10.1192/j.eurpsy.2021.200

**Published:** 2021-08-13

**Authors:** K. Krysta

**Affiliations:** Department Of Rehabilitation Psychiatry, Medical University of Silesia, Katowice, Poland

**Keywords:** intellectual disability, Functioning, diagnosis

## Abstract

The introduction of the ICF scale in Poland started a few years ago. One of the institutions, which started trainings and implementation of the scale is the Polish Association for Persons with Intellectual Disability (PSONI). The use of the ICF scale turned out to be very important because the diagnosis itself still tells us little about the life and personality of a person. The disorder itself does not determine functioning in society. The application of this scale helps to capture the dynamics between concepts such as health, human body functions, ability to participate in different activities, environmental factors and personality. It allows, for example, to clearly define whether the problems are caused by the health of a given person or by external limitations, even of an architectural nature. It is expected to implement ICF in different centers run by the Association like e.g. Vocational Activity Centers or the Center for Vocational Counseling and Support for People with Intellectual Disabilities (DZWONI). The scale is also being implemented in Health Care Services for Individuals with ID and Mental Health Problems in Poland because it remains an integrative model and not only medical and social one.

**Disclosure:**

No significant relationships.

